# Clinical Impact of Thrombus Aspiration and Interaction With D-Dimer Levels in Patients With ST-Segment Elevation Myocardial Infarction Undergoing Primary Percutaneous Coronary Intervention

**DOI:** 10.3389/fcvm.2021.706979

**Published:** 2021-08-10

**Authors:** Jun-feng Li, Zhi-wei Lin, Chang-xi Chen, Shi-qi Liang, Lei-lei Du, Xiang Qu, Zhan Gao, Yu-heng Huang, Shu-ting Kong, Jin-xin Chen, Ling-yue Sun, Hao Zhou

**Affiliations:** ^1^Department of Cardiology, The First Affiliated Hospital of Wenzhou Medical University, Wenzhou, China; ^2^Department of Cardiology, Ren Ji Hospital, School of Medicine, Shanghai Jiao Tong University, Shanghai, China

**Keywords:** thrombus aspiration, D-D, percutaneous coronary intervention, STEMI, coronary artery disease

## Abstract

**Objectives:** To evaluate the effect of thrombus aspiration (TA) strategy on the outcomes and its interaction with D-dimer levels in patients with ST-segment elevation myocardial infarction (STEMI) during primary percutaneous coronary intervention (PCI) in “real-world” settings.

**Materials and Methods:** This study included 1,295 patients with STEMI who had undergone primary PCI with or without TA between January 2013 and June 2017. Patients were first divided into a TA+PCI group and a PCI-only group, and the baseline characteristics and long-term mortality between the two groups were analyzed. Furthermore, we studied the effect of TA on the clinical outcomes of patients grouped according to quartiles of respective D-dimer levels. The primary outcome was all-cause mortality, and the secondary outcomes were new-onset heart failure (HF), rehospitalization, re-PCI, and stroke.

**Results:** In the original cohort, there were no significant differences in all-cause mortality between the TA+PCI and PCI-only groups (hazard ratio, 0.789; 95% confidence interval, 0.556–1.120; *p* = 0.185). After a mean follow-up of 2.5 years, the all-cause mortality rates of patients in the TA + PCI and PCI-only groups were 8.5 and 16.2%, respectively. Additionally, differences between the two groups in terms of the risk of HF, re-PCI, rehospitalization, and stroke were non-significant. However, after dividing into quartiles, as the D-dimer levels increased, the all-cause mortality rate in the PCI group gradually increased (4.3 vs. 6.0 vs. 7.0 vs. 14.7%, *p* < 0.001), while the death rate in the TA+PCI group did not significantly differ (4.6 vs. 5.0 vs. 4.0 vs. 3.75%, *p* = 0.85). Besides, in the quartile 3 (Q3) and quartile 4 (Q4) groups, the PCI-only group was associated with a higher risk of all-cause mortality than that of the TA+PCI group (Q3: 4.0 vs. 7.0%, *p* = 0.029; Q4: 3.75 vs. 14.7%, *p* < 0.001). Moreover, the multivariate logistic regression analysis demonstrated that TA is inversely associated with the primary outcome in the Q4 group [odds ratio (OR), 0.395; 95% CI, 0.164–0.949; *p* = 0.038].

**Conclusions:** The findings of our real-world study express that routine manual TA during PCI in STEMI did not improve clinical outcomes overall. However, patients with STEMI with a higher concentration of D-dimer might benefit from the use of TA during primary PCI. Large-scale studies are recommended to confirm the efficacy of TA.

## Introduction

Prompt reperfusion therapy has been recommended as the first and best treatment in patients with acute ST-segment elevation myocardial infarction (STEMI) (1, 2). Primary percutaneous coronary intervention (PCI) is the most optimal and effective treatment in patients with STEMI. Coronary thrombosis is a hallmark of acute STEMI. Thrombus burden and reduced myocardial perfusion are essential predictors of poor clinical outcomes (3). Although thrombus aspiration (TA) has been considered one of the reperfusion strategies and is widely implemented in primary PCI, its benefits are debatable (1, 4). The benefits of TA have not been consistently demonstrated in previous clinical trials. Preliminary studies have confirmed that TA during primary PCI has clinical relevance compared with conventional primary PCI (5–8). However, recent major clinical randomized controlled trials (TASTE and TOTAL trials) have challenged the clinical benefits of TA and do not support the routine use of manual TA in patients with STEMI requiring primary PCI (9, 10). Due to such conflicting results, the routine use of TA during primary PCI is not recommended (1).

Given the pathophysiology of acute STEMI involving interactions among ruptured plaque-induced coronary thrombi and platelets and clotting factors (11, 12), the D-dimer level, a useful and direct biomarker of crosslinked procoagulant activity and ongoing fibrinolysis, has been associated with acute STEMI (13, 14). In these patients, elevated D-dimer levels at the time of admission were associated with increased cardiovascular mortality and all-cause mortality following primary PCI. Even in healthy individuals, the D-dimer level has been proven to be a predictor of cardiovascular events (15). To the best of our knowledge, the association between D-dimer levels and long-term outcomes in patients with STEMI undergoing TA during primary PCI has not been evaluated. Further studies are required to clarify whether patients with high D-dimer levels stand to benefit the most from TA.

To date, real-world data regarding TA and its effect in patients with STEMI have been conflicting (4, 16). Our study aimed to investigate the effect of TA on the clinical outcomes and prognoses and its relationship with different D-dimer levels in patients with STEMI undergoing primary PCI.

## Materials and Methods

### Study Design and Patient Enrollment

This single-center study compared the effect of manual TA before PCI with PCI only on clinical outcomes and prognoses in patients with STEMI. A total of 1,295 consecutive patients were enrolled between January 2013 and June 2016 and were followed up to June 2017. The patients were divided into two groups based on treatment with manual TA before PCI (TA+PCI group) or PCI alone (PCI-only group). Patient inclusion was based on ≥18 years of age, STEMI diagnosis in ≤ 12 h of symptom onset, and angiographically determined Thrombolysis in Myocardial Infarction flow grade of 0–1. According to the European Society of Cardiology (ESC) guidelines, STEMI was defined as chest pain of more than 30 min suggestive of acute myocardial ischemia prior to hospital admission and an electrocardiogram reading ≥0.1 mV for the ST-segment elevation in two or more contiguous leads, new left bundle branch block (LBBB), or new-onset Q waves (1). Patients were excluded in case of the following: (1) contraindications to contrast agents and therapeutic medications, (2) history of coronary artery bypass surgery, (3) preoperative cardiogenic shock, (4) received fibrinolytic therapy, (5) life expectancy <6 months, (6) recent major surgery or trauma, and (7) unwilling or unable to provide informed consent.

Before primary PCI, pharmacological pretreatment comprised the administration of aspirin and clopidogrel or ticagrelor. During the primary PCI procedure, unless contraindicated, patients received a glycoprotein IIb/IIIa inhibitor (tirofiban) according to their weight and additional unfractionated heparin to achieve an activated clotting time of 250–300 s. Following primary PCI, the standard dual antiplatelet therapy comprised aspirin, clopidogrel, or ticagrelor for at least 12 months after stent implantation. Other pharmacological treatments, including β-blockers, statins, angiotensin-converting enzyme inhibitors (ACEIs) or angiotensin II receptor blockers (ARBs), and diuretics, if necessary, were recommended based on current guidelines.

In this cohort study, the decision for manual TA was based on the operator's discretion, and it was performed according to the standard protocol. Thrombus grade was classified according to Gibson et al. (17): grade 0, no angiographic characteristics of thrombus are present; grade 1, possible thrombus, with angiographic characteristics such as reduced contrast density, haziness, irregular lesion contour, or a smooth convex “meniscus” at the site of total occlusion suggestive but not diagnostic of thrombus; grade 2, definite thrombus, with the greatest dimension less than or equal to half of the vessel diameter; grade 3, definite thrombus but with the greatest linear dimension greater than half but less than twice the vessel diameter; grade 4, definite thrombus, with the largest dimension greater than or equal to twice the vessel diameter; and grade 5, total occlusion. The procedure was performed by investigators who were experienced interventional doctors. Investigators guided the catheter in place, sent the guidewire through the lesion, and then pushed the thrombectomy catheter. When the thrombectomy catheter reached the proximal part of the thrombus along the guidewire, suction was performed under negative pressure. Following aspiration, the guide catheter was confirmed to have entered the coronary ostium and the thrombectomy catheter was withdrawn under negative pressure.

### Clinical Laboratory Data Collection

Baseline demographics, clinical presentations, laboratory data, and echocardiography findings were collected from the medical records. Laboratory data during hospitalization, including peak troponin I, peak creatine kinase isoenzyme MB (CK-MB), brain natriuretic peptide (BNP), creatinine (Cr), low-density lipoprotein cholesterol, lactic acid, glucose, C-reactive protein, and D-dimer levels, were recorded before primary PCI, and laboratory data, including D-dimer levels, were collected as soon as emergency patients were admitted to the hospital, which was strictly controlled and recorded. This study was approved by the local ethics committee. All participants provided written informed consent. The study protocol was approved by the local research ethics board.

### Follow-Up and Outcome Measures

All enrolled participants were asked to return for a regular outpatient follow-up at 1 month and then each year after discharge. Clinical follow-up was conducted for all enrolled patients to evaluate the occurrence of the following major adverse cardiac events: death, non-fatal reinfarction, target vessel revascularization, and heart failure (HF). The primary endpoint was all-cause mortality during long-term follow-up, and secondary endpoints were new-onset HF, rehospitalization for coronary heart disease (including reinfarction, stent thrombosis, and target vessel revascularization), re-PCI, and stroke. Major adverse events were assessed up to June 30, 2017, and patient follow-up was censored at the time of death. Follow-up data were collected from outpatient visit or telephone call. The median follow-up duration was 2.5 years.

### Statistical Analysis

Baseline characteristics of patients with and without TA are described as mean ± standard deviation (SD) for all continuous variables, and non-normally distributed parameters are shown as median (25th and 75th percentile). Data for categorical variables are reported as values and percentages and were compared using the chi-square test or Fisher's exact test. Comparison of continuous variables between the two groups was performed using Student's *t*-test and analysis of variance for multiple comparisons. The Mann–Whitney *U*-test was used for non-normally distributed variables. All analyses were based on the intention-to-treat principle.

First, univariable analyses were performed to determine the difference between the two groups in the primary and secondary outcomes and its components (HF, rehospitalization, re-PCI, and stroke). Kaplan–Meier curves were generated to create event-free survival curves, and the log-rank test was used to compare the distributions of time-to-event during the follow-up. Second, multivariable Cox proportional hazards analyses were performed to examine the association between TA and all-cause mortality in each group. For statistical adjustment, we included the following variables: age; sex; stent; TA; multivessel disease; medical therapy with aspirin, ACEIs, metoprolol, spironolactone; laboratory data regarding lactic acid, BNP, and Cr; left ventricular ejection fraction (LVEF); and history of smoking and diabetes mellitus. Non-normally distributed variables were included in the multivariate regression model after logarithmic transformation. Hazard ratios (HRs), 95% confidence intervals (CIs), and *p*-values were calculated.

To further investigate the interaction of TA with D-dimer levels, patients were categorized into four groups (quartiles) according to the D-dimer levels. The primary and secondary outcomes were analyzed and compared based on whether TA was performed. Multivariable logistic regression models were used to determine independent predictors of all-cause mortality.

All analyses were conducted using SPSS version 25 (IBM Corporation, USA). Calculations were performed using MedCalc 12.9 statistical software (Mariakerke, Belgium). A value of *p* < 0.05 in the two-tailed test was considered statistically significant.

## Results

### Patient Characteristics

From January 2013 to July 2016, a total of 1,295 patients who met our inclusion criteria were prospectively enrolled to undergo primary PCI during the study period. Of these, 657 (50.7%) underwent PCI with TA and 638 (49.3%) underwent only PCI. Differences in the baseline characteristics were significant between the TA+PCI group and PCI-only group (Table 1). Patients in the TA + PCI group were younger; more likely to be smokers; had higher glucose, troponin I, and CK-MB levels; and had lower Cr levels and LVEF compared with those of the PCI-only group. Additionally, patients in the TA + PCI group were more often treated with aspirin, clopidogrel, statins, and β-blockers than those in the PCI-only group. In contrast, patients in the PCI-only group were more likely to have multivessel disease and less likely to have a stent implantation, and the culprit artery was more frequently the proximal left anterior descending artery compared with patients in the TA+PCI group.

**Table 1:** Baseline clinical and angiographic characteristics of all enrolled patients.

**Characteristics**	**All enrolled patients**		
	**TA + PCI (657)**	**PCI-only (638)**	***P*-value**
**Demographic data**			
Age, years	62.2 ± 12.8	65.7 ± 12.8	<0.001
Male, *n* (%)	520 (79.1)	499 (78.2)	0.681
**Medical history**, ***n*****(%)**			
Smoker	282 (42.9)	234 (36.7)	0.022
Alcohol	112 (17.0)	89 (13.9)	0.124
Previous MI	7 (1.1)	16 (2.5)	0.049
Hypertension	344 (52.4)	369 (57.8)	0.048
Diabetes	135 (20.5)	152 (23.8)	0.156
Hyperlipidemia	105 (16.0)	101 (15.8)	0.941
**Laboratory measurements**			
Troponin I, μg/L	5.60 (0.42–50.0)	3.79 (0.52–27.89)	0.034
CK-MB, U/L	77.0 (24.0–252.0)	55.0 (24.0–149.0)	0.001
BNP, pg/mL	229.0 (99.0–652.0)	317.0 (101.0–922.0)	0.08
Creatinine, μmoI/L	70.0 (58.0–85.0)	73.0 (60.0–90.0)	0.012
LDL-C, mmol/L	3.17 ± 1.10	3.13 ± 1.00	0.463
Lactic acid, mmol/L	2.20 (1.60–3.30)	2.20 (1.60–3.23)	0.554
Glucose, mmol/L	7.20 (6.20–9.00)	6.80 (5.50–8.80)	0.016
C-reactive protein, mg/L	9.80 (5.20–26.00)	10.40 (5.03–29.70)	0.574
**Echocardiographic data**			
LVEF, (%)	48.6 ± 9.3	50.7 ± 10.8	<0.001
**Medications**, ***n*****(%)**			
Aspirin	611 (93.0)	534 (83.7)	<0.001
Clopidogrel	599 (91.2)	520 (81.5)	<0.001
Ticagrelor	13 (2.0)	9 (1.4)	0.429
Statins	600 (91.3)	529 (82.9)	<0.001
ACEI or ARB	361 (54.9)	345 (54.1)	0.919
β-blocker	448 (68.2)	365 (57.2)	<0.001
Aldosterone antagonist	191 (29.1)	16 (25.9)	0.196
Other diuretics	191 (29.1)	180 (28.2)	0.733
**Percutaneous coronary intervention**, ***n*****(%)**			
Multivessel disease	345 (52.5)	392 (61.4)	0.001
Proximal LAD artery	326 (49.6)	280 (43.9)	0.039
Stent	579 (88.1)	492 (77.1)	<0.001

*TA, thrombus aspiration; PCI, percutaneous coronary intervention; MI, myocardial infarction; CK-MB, creatine kinase isoenzyme MB; BNP, brain natriuretic peptide; LDL-C, low-density lipoprotein cholesterol; LVEF, left ventricular ejection fraction; ACEI, angiotensin-converting–enzyme inhibitors; ARB, angiotensin-II–receptor blockers; IABP, intra-aortic balloon pump; LAD, left anterior descending*.

After stratification by quartiles based on D-dimer levels, four groups of patients were obtained. Table 2 presents the baseline characteristics of patients stratified according to the D-dimer levels. The first quartile (Q1; D-dimer ≤ 0.54, *n* = 329), second quartile (Q2; 0.54 < D-dimer ≤ 0.91, *n* = 319), third quartile (Q3; 0.91 < D-dimer ≤ 1.50, *n* = 327), and fourth quartile (Q4; 1.50 < D-dimer, *n* = 320). With increasing D-dimer levels, patients were observed to be older, with a history of drinking, and with greater susceptibility to multivessel disease.

**Table 2:** Baseline characteristics according to plasma D-dimer concentrations and treatment group.

**Characteristics**	**D-dimer quartile 1**		**D-dimer quartile 2**		**D-dimer quartile 3**		**D-dimer quartile 4**		***P*-value**
	**≤0.54 (** ***n*** **=** **329)**		**0.54<**. **≤0.91 (*****n*****=319)**		**0.91<**. **≤1.50 (*****n*****=327)**		**1.5 < ** **(** ***n*** **=** **320)**		
	**TA + PCI**	**PCI-only**	**TA + PCI**	**PCI-only**	**TA + PCI**	**PCI-only**	**TA + PCI**	**PCI-only**	
	**(*n* = 173)**	**(*n* = 156)**	**(*n* = 159)**	**(*n* = 160)**	**(*n* = 174)**	**(*n* = 153)**	**(*n* = 151)**	**(*n* =169)**	
**Demographic data**									
Age, years	61.8 ± 13.3	63.8 ± 13.2	61.1 ± 12.9	62.6 ± 12.6	63.0 ± 12.3	67.1 ± 12.1	62.8 ± 12.9	69.2 ± 12.1	*P*_56_ = 0.002, *P*_78_ <0.01
Male, *n* (%)	132 (76.3)	130 (83.3)	136 (85.5)	126 (78.8)	139 (79.9)	121 (79.1)	113 (74.8)	122 (72.2)	NS
**Medical history**, ***n*****(%)**									
Smoker	75 (43.4)	60 (38.5)	69 (43.4)	69 (43.1)	80 (46.0)	53 (34.6)	58 (38.4)	52 (30.8)	*P*_56_ = 0.037
Alcohol	26 (15.0)	18 (11.5)	26 (16.4)	23 (14.4)	31 (17.8)	22 (14.4)	29 (19.2)	26 (15.4)	NS
Previous MI	2 (1.2)	2 (1.3)	5 (3.1)	6 (3.8)	0	6 (3.9)	0	2 (1.2)	*P*_56_ = 0.026
Hypertension	81 (46.8)	77 (49.4)	79 (49.7)	92 (57.5)	104 (59.8)	99 (64.1)	80 (53.0)	102 (60.4)	NS
Diabetes	33 (19.1)	31 (19.9)	29 (18.2)	45 (28.1)	32 (18.4)	32 (20.9)	41 (27.2)	44 (26.0)	*P*_34_ = 0.036
Hyperlipidemia	36 (20.8)	25 (16.0)	31 (19.5)	32 (20.0)	25 (14.4)	24 (15.7)	13 (8.6)	20 (11.8)	NS
**Laboratory measurements**									
Troponin I, μg/L	6.15 (0.23–50.0)	3.15 (0.51–18.5)	2.78 (0.36–50.0)	2.54 (0.39–19.7)	5.76 (0.54–50.0)	5.67 (0.73–40.25)	22.72 (0.85–50.0)	4.43 (0.85–50.0)	*P*_78_ = 0.046
CK-MB, U/L	73.0 (19.0–269.75)	53.5 (24.0–157.0)	45.0 (25.0–228.0)	45.0 (20.0–128.75)	76.5 (28.75–215.75)	75.0 (22.0–192.5)	90.0 (27.0–337.0)	56.0 (24.5–133.5)	*P*_34_ = 0.022, *P*_78_ = 0.008
BNP, pg/mL	235.5 (83.25–695.0)	208.5 (92.75–585.0)	195.0 (96.0–478.0)	166.5 (78.0–475.0)	199.5 (97.50–540.75)	361.0 (103.5–1157.5)	399.0 (119.0–948.0)	724.5 (190.75–2364.5)	*P*_56_ = 0.011, *P*_78_ = 0.001
Creatinine, μmoI/L	68.5 (57.0–80.75)	71.0 (59.0–85.0)	69.0 (60.0–81.0)	68.0 (58.0–82.75)	71.0 (59.75–88.0)	73.0 (60.0–88.0)	71.0 (57.0–93.0)	82.0 (64.0–115.0)	*P*_78_ = 0.003
LDL-C, mmol/L	3.32 ± 1.16	3.23 ± 1.03	3.14 ± 1.06	3.15 ± 0.96	3.17 ± 1.12	3.17 ± 1.05	3.02 ± 1.06	3.00 ± 0.93	NS
Lactic acid, mmol/L	2.10 (1.65–3.20)	2.20 (1.60–3.10)	2.10 (1.60–3.10)	2.20 (1.60–2.90)	2.25 (1.60–3.23)	2.20 (1.50–2.90)	2.40 (1.60–3.70)	2.50 (1.65–4.75)	NS
Glucose, mmol/L	7.40 (6.30–9.30)	6.30 (5.30–8.08)	6.60 (5.90–8.50)	6.70 (5.53–8.40)	7.10 (6.20–8.93)	7.0 (5.75–8.85)	7.60 (6.20–9.90)	7.20 (5.50–10.0)	*P*_12_ = 0.001
C-reactive protein, mg/L	9.60 (6.01–34.98)	13.45 (5.80–33.10)	7.80 (5.0–15.10)	8.30 (5.0–20.50)	9.46 (5.53–21.98)	8.35 (5.0–16.20)	19.0 (8.0–47.50)	21.10 (7.65–65.70)	NS
**Echocardiographic data**									
LVEF, (%)	48.8 ± 9.6	52.2 ± 9.2	48.7 ± 9.2	53.1 ± 11.0	48.6 ± 10.0	50.0 ± 10.7	48.5 ± 8.3	48.0 ± 11.3	*P*_12_ = 0.001, *P*_34_ <0.01
**Medications**, ***n*****(%)**									
Aspirin	155 (89.6)	127 (81.4)	155 (97.5)	145 (90.6)	163 (93.7)	135 (88.2)	138 (91.4)	127 (75.1)	*P*_12_ = 0.02, *P*_34_ = 0.01, *P*_78_ <0.01
Clopidogrel	157 (90.8)	129 (82.7)	154 (96.9)	139 (86.9)	152 (87.4)	128 (83.7)	136 (90.1)	124 (73.4)	
Ticagrelor	2 (1.2)	1 (0.6)	3 (1.9)	2 (1.3)	5 (2.9)	6 (3.9)	3 (2.0)	0	NS
Statins	156 (90.2)	129 (82.7)	152 (95.6)	146 (91.3)	158 (90.8)	135 (88.3)	134 (88.8)	119 (70.4)	NS
ACEI or ARB	85 (49.1)	82 (52.6)	88 (55.4)	98 (61.3)	104 (59.8)	90 (58.8)	84 (55.6)	75 (44.4)	NS
β-blocker	113 (65.3)	89 (57.1)	114 (71.7)	101 (63.1)	122 (70.1)	89 (58.2)	99 (65.6)	86 (50.9)	*P*_56_ = 0.024, *P*_78_ = 0.008
Aldosterone antagonist	53 (30.6)	39 (25.0)	39 (24.5)	36 (22.5)	51 (29.3)	41 (26.8)	48 (31.8)	49 (29.0)	NS
Other diuretics	54 (31.2)	39 (25.0)	39 (24.5)	39 (24.4)	50 (28.7)	45 (29.4)	48 (31.8)	57 (33.7)	NS
**PCI**, ***n*****(%)**									
Multi	90 (52.0)	87 (55.8)	69 (43.4)	90 (56.3)	99 (56.9)	107 (69.9)	87 (57.6)	108 (63.9)	*P*_34_ = 0.022, *P*_56_ = 0.015
Proximal LAD artery	94 (54.3)	52 (33.3)	74 (46.5)	72 (45.0)	89 (51.1)	80 (52.3)	69 (45.7)	76 (45.0)	*P*_12_ <0.01
Stent	157 (90.8)	114 (73.1)	138 (86.8)	129 (80.6)	149 (85.6)	126 (82.4)	135 (89.4)	123 (72.8)	*P*_12_ <0.01, *P*_78_ <0.01

*TA, thrombus aspiration; PCI, percutaneous coronary intervention; MI, myocardial infarction; CK-MB, creatine kinase isoenzyme MB; BNP, brain natriuretic peptide; LDL-C, low-density lipoprotein cholesterol; LVEF, left ventricular ejection fraction; ACEI, angiotensin-converting–enzyme inhibitors; ARB, angiotensin-II–receptor blockers; IABP, intra-aortic balloon pump; LAD, left anterior descending; NS, no significance; *P*_12_, *P*-value between the TA + PCI and PCI-only in group Quartile 1; *P*_34_, *P*-value between the TA + PCI and PCI-only in group Quartile 2; *P*_56_, *P*-value between the TA + PCI and PCI-only in group Quartile 3; *P*_78_, *P*-value between the TA + PCI and PCI-only in group Quartile 4.*

### Primary and Secondary Outcomes

**Figure 1:**
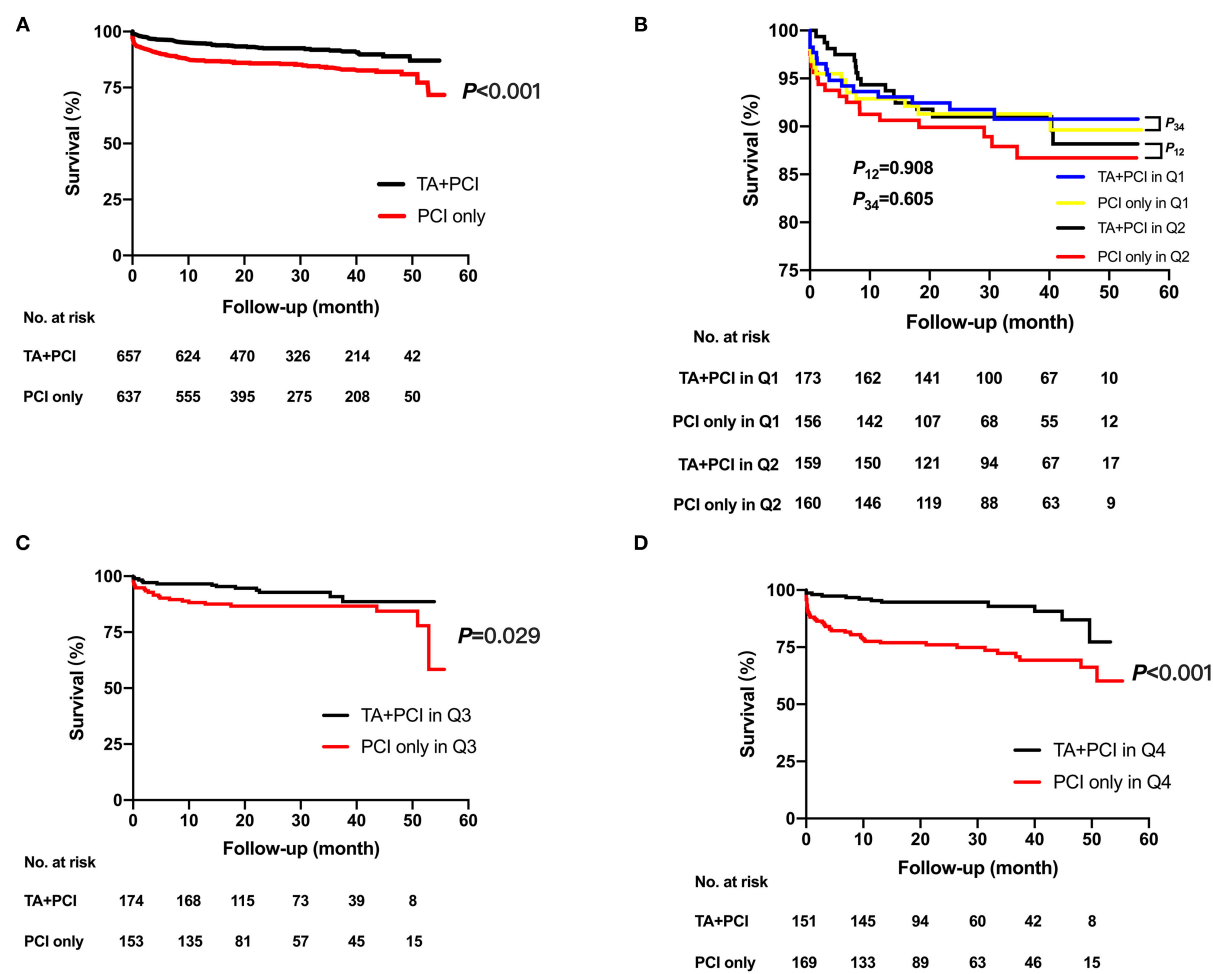
Kaplan–Meier time-to-event curves for all-cause death for the total duration of follow-up after the intervention. Kaplan–Meier estimates in TA + PCI group (red curve) and PCI-only group (black curve) for the rate of all-cause mortality in the whole population **(A)** and all-cause mortality of TA + PCI group (blue curve) and PCI-only group (yellow curve) in quartile 1; all-cause mortality of TA + PCI group (black curve) and PCI-only group (red curve) in quartile 2 **(B)**; all-cause mortality of TA + PCI group (black curve) and PCI-only group (red curve) in quartile 3 **(C)**; and all-cause mortality of TA + PCI group (black curve) and PCI-only group (red curve) in quartile 4 **(D)**. TA, thrombus aspiration; PCI, percutaneous coronary intervention; *P*12, *p*-value between the TA + PCI and PCI-only in group Quartile 1; *P*34, *p*-value between the TA + PCI and PCI-only in group Quartile 2.

The primary and secondary endpoints in the TA + PCI and PCI-only groups are shown in Table 3 and Figure 1A. In the univariate analysis, the all-cause mortality in the TA + PCI group (8.5%) was lower than that in the PCI-only group (16.2%) (3.40 vs. 6.47% per year; HR, 0.502; 95% CI, 0.362–0.695; *p* < 0.001). However, the difference between the two groups was not significant in terms of the risk of HF (2.66 vs. 2.15% per year; HR, 1.369; 95% CI, 0.847–2.211; *p* = 0.200), re-PCI (1.86 vs. 1.93% per year; HR, 1.194; 95% CI, 0.687–2.075; *p* = 0.529), rehospitalization (4.27 vs. 3.64% per year; HR, 1.343; 95% CI, 0.918–1.965; *p* = 0.129), and stroke (0.13 vs. 0.15% per year; HR, 0.899; 95% CI, 0.126–6.401; *p* = 0.916). This seems to mean that emergency PCI patients can benefit from TA, thereby reducing long-term mortality. Table 4 presents the stepwise multivariate Cox regression analysis of the association between TA and all-cause mortality. Factors found to have predictive significance (*p* < 0.05) in the univariate analysis between the two groups were included in the multivariate regression model. In the multivariate analysis, no interaction with TA was noted with all-cause mortality (*p* = 0.185; HR, 0.789; 95% CI, 0.556–1.120), whereas sex, age, stent implantation, multiple vessels, administration of aspirin, BNP, and LVEF demonstrated a considerable interaction with the primary outcome.

**Table 3:** Primary and secondary end points in the all enrolled patients.

**Outcome**	**No. of patients with event *n* (%)**	**Event rate (%/ys)**	**Hazard ratio (95% CI)**	***P*-value**
**All-cause death**				
TA + PCI	56 (8.5)	3.40	0.502 (0.362–0.695)	<0.001
PCI-only	103 (16.2)	6.47	Reference	
**HF**				
TA + PCI	40 (6.7)	2.66	1.369 (0.847–2.211)	0.200
PCI-only	29 (5.4)	2.15	Reference	
**Re-PCI**				
TA + PCI	28 (4.7)	1.86	1.194(0.687–2.075)	0.529
PCI-only	26 (4.8)	1.93	Reference	
**Re-hospitalization**				
TA + PCI	64 (10.7)	4.27	1.343 (0.918–1.965)	0.129
PCI-only	49 (9.1)	3.64	Reference	
**Stroke**				
TA + PCI	2 (0.3)	0.13	0.899 (0.126–6.401)	0.916
PCI-only	2 (0.4)	0.15	Reference	

*TA, thrombus aspiration; PCI, percutaneous coronary intervention*.

**Table 4:** Multivariate Cox regression analyses of all enrolled patients.

**Variables**	**All-cause death**		
	**Hazard**	**95% confidence**	*P*-value^*^ (*p*)
	**ratio (HR)**	**interval (95% CI)**	
Sex	0.655	0.454–0.974	0.036
Age	1.054	1.037–1.072	<0.001
Stent	0.642	0.442–0.933	0.020
Thrombus aspiration	0.789	0.556–1.120	0.185
Multi	1.518	1.057–2.178	0.024
Aspirin	0.353	0.224–0.555	<0.001
ACEI	0.814	0.538–1.232	0.330
Metoprolol	0.777	0.534–1.130	0.186
Spironolactone	1.048	0.721–1.523	0.807
Lactic acid	0.976	0.744–1.280	0.859
BNP	1.220	1.058–1.407	0.006
Creatinine	1.170	0.824–1.661	0.379
LVEF	0.969	0.952–0.985	<0.001
Diabetes	1.363	0.951–1.955	0.092
Smoker	1.447	0.952–2.199	0.084

*TA, thrombus aspiration; PCI, percutaneous coronary intervention; MI, myocardial infarction; CK-MB, creatine kinase isoenzyme MB; BNP, brain natriuretic peptide; LDL-C, low-density lipoprotein cholesterol; LVEF, left ventricular ejection fraction; ACEI, angiotensin-converting–enzyme inhibitors; ARB, angiotensin-II–receptor blockers; IABP, intra-aortic balloon pump; LAD, left anterior descending*.

* *Values were obtained by multivariate analysis after adjustment for age, gender, stent, thrombus aspiration, multivessel disease, medical therapy of aspirin, ACEI, β-blocker (metoprolol), spironolactone, laboratory data of lactic acid (Lac), brain natriuretic peptide (BNP), creatinine (Cr), ejection fraction (LVEF), history of smoking and diabetes mellitus (DM)*.

To determine the association between TA and D-dimer levels, the primary and secondary endpoints were calculated in patients stratified by quartiles of D-dimer levels. Multivariate logistic regression models were used to determine independent predictors of all-cause mortality. The results showed that as the levels of D-dimer increased, the all-cause mortality rates gradually increased in patients who had undergone only PCI (4.3 vs. 6.0 vs. 7.0 vs. 14.7%, *p* < 0.001). However, the mortality rates in patients who underwent TA + PCI did not change significantly (4.6 vs. 5.0 vs. 4.0 vs. 3.75%, *p* = 0.85). Specifically, patients in Q4 had the worst outcomes following PCI. At the same time, patients in the PCI-only group were associated with a higher all-cause mortality than those in the TA+PCI group in Q3 and Q4 (Q3: 4.0 vs. 7.0%, *p* = 0.029; Q4: 3.75 vs. 14.7%, *p* < 0.001) (Table 5; Figures 1B–D).

**Table 5:** Primary and secondary end points for the subgroup analysis with different levels of D-dimer.

**Outcome**	**D-dimer quartile 1**		**D-dimer quartile 2**		**D-dimer quartile 3**		**D-dimer quartile 4**		***P*-value**
	**≤0.54 (** ***n*** **=** **329)**		**0.54<**. **≤0.91 (*****n*****=319)**		**0.91<**. **≤1.50 (*****n*****=327)**		**1.5 < ** **(** ***n*** **=** **320)**		
	**TA + PCI**	**PCI-only**	**TA + PCI**	**PCI-only**	**TA + PCI**	**PCI-only**	**TA + PCI**	**PCI-only**	
All-cause death, *n* (%)	15 (4.6%)	14(4.3%)	16 (5.0%)	19 (6.0%)	13 (4.0%)	23 (7.0%)	12 (3.75%)	47 (14.7%)	*P*_12_ = 0.908, *P*_34_ = 0.605, *P*_56_ = 0.029, *P*_78_ <0.001
HF, *n* (%)	9 (2.7%)	7 (2.1%)	12 (3.8%)	8 (2.5%)	8 (2.4%)	5 (1.5%)	11 (3.4%)	9 (2.8%)	NS
Re-PCI, *n* (%)	4 (1.2%)	3 (0.9%)	9 (2.8%)	6 (1.9%)	11 (3.4%)	7 (2.1%)	4 (1.25%)	10 (3.1%)	NS
Re-hospitalization, *n* (%)	13 (4.0%)	7 (2.1%)	15 (4.7%)	11 (3.4%)	21 (6.4%)	15 (4.6%)	15 (4.7%)	16 (5%)	NS
Stroke	1 (0.3%)	1 (0.3%)	1 (0.3%)	0	0	1 (0.3%)	0	0	NS

*TA, thrombus aspiration; PCI, percutaneous coronary intervention; HF, heart failure; NS, no significance; P_12_, P-value between the TA + PCI and PCI-only in group Quartile 1; P_34_, P-value between the TA + PCI and PCI-only in group Quartile 2; P_56_, P-value between the TA + PCI and PCI-only in group Quartile 3; P_78_, P-value between the TA + PCI and PCI-only in group Quartile 4*.

Moreover, the results of multivariate logistic regression analysis for all-cause mortality, which was used to investigate the role of TA for the different D-dimer quartiles, are given in Table 6. In Q4 (D-dimer >1.5), TA was inversely associated with the primary outcome, which indicated that TA was an independent predictor for all-cause mortality in patients with high D-dimer levels (OR, 0.395; 95% CI, 0.164–0.949; *p* = 0.038). No significant association between TA and all-cause mortality was observed in the first three groups (Q1: OR, 1.446; 95% CI, 0.537–3.897; *p* = 0.466; Q2: OR, 1.304; 95% CI, 0.518–3.287; *p* = 0.573; and Q3: OR, 0.779; 95% CI, 0.314–1.932; *p* = 0.590).

**Table 6:** Multivariable logistic regression analyses according to quartile of D-dimer levels.

**Variables odds ratio; 95% confidence interval; *P*-value**	**All-cause death quarted by D-dimer level (mg/L)**			
	**Quartile 1**	**Quartile 2**	**Quartile 3**	**Quartile 4**
	**≤0.54**	**0.54 < ... ≤ 0.91**	**0.91 < ... ≤ 1.50**	**1.5 < **
Sex	0.197 (0.056–0.694) 0.011	0.391 (0.118–1.297) 0.125	1.971 (0.632–6.147) 0.242	0.445 (0.170–1.163) 0.098
Age	1.047 (0.998–1.098) 0.063	1.086 (1.035–1.139) 0.001	1.079 (1.029–1.132) 0.002	1.051 (1.011–1.094) 0.012
Stent	0.453 (0.151–1.361) 0.158	2.123 (0.595–7.583) 0.246	0.177 (0.064–0.491) 0.01	0.904 (0.353–2.318) 0.834
Thrombus aspiration	1.446 (0.537–3.897) 0.466	1.304 (0.518–3.287) 0.573	0.779 (0.314–1.932) 0.590	0.395 (0.164–0.949) 0.038
Multi	3.228 (1.045–9.974) 0.042	1.360 (0.552–3.350) 0.504	0.908 (0.356–2.317) 0.840	1.765 (0.748–4.163) 0.195
Aspirin	0.818 (0.197–3.397) 0.782	0.052 (0.009–0.301) 0.001	0.537 (0.131–2.202) 0.388	0.168 (0.061–0.464) 0.001
ACEI	0.716 (0.239–2.144) 0.551	0.416 (0.147–1.179) 0.099	3.073 (1.098–8.596) 0.032	0.246 (0.074–0.819) 0.022
Metoprolol	1.302 (0.454–3.730) 0.623	0.704 (0.282–1.756) 0.452	0.992 (0.342–2.872) 0.988	0.514 (0.212–1.245) 0.140
Spironolactone	0.294 (0.089–0.976) 0.046	3.024 (1.088–8.407) 0.034	1.538 (0.564–4.190) 0.400	1.034 (0.434–2.461) 0.941
Lactic acid	1.314 (0.531–3.251) 0.554	0.933 (0.345–2.526) 0.892	1.951 (0.789–4.824) 0.148	0.788 (0.418–1.484) 0.460
BNP	2.442 (1.486–4.013) 0.001	0.999 (0.639–1.563) 0.998	1.494 (1.016–2.197) 0.041	0.925 (0.679–1.260) 0.620
Creatinine	2.010 (0.497–8.125) 0.327	2.113 (0.691–6.459) 0.189	1.040 (0.396–2.728) 0.937	3.673 (1.423–9.481) 0.007
LVEF	0.963 (0.914–1.014) 0.150	0.970 (0.927–1.015) 0.184	0.979 (0.935–1.025) 0.367	0.942 (0.904–0.982) 0.005
Diabetes	0.566 (0159–2.009) 0.378	2.828 (1.058–7.559) 0.038	2.061 (0.768–5.529) 0.151	1.372 (0.566–3.327) 0.484
Smoker	3.861 (0.905–16.473) 0.068	1.552 (0.509–4.729) 0.440	1.395 (0.491–3.967) 0.532	1.978 (0.756–5.173) 0.164

*TA, thrombus aspiration; PCI, percutaneous coronary intervention; MI, myocardial infarction; CK-MB, creatine kinase isoenzyme MB; BNP, brain natriuretic peptide; LDL-C, low-density lipoprotein cholesterol; LVEF, left ventricular ejection fraction; ACEI, angiotensin-converting–enzyme inhibitors; ARB, angiotensin-II–receptor blockers; IABP, intra-aortic balloon pump; LAD, left anterior descending*.

*Values were obtained by multivariate analysis after adjustment for age, gender, stent, thrombus aspiration, multivessel disease, medical therapy of aspirin, ACEI, β-blocker (metoprolol), spironolactone, laboratory data of lactic acid (Lac), brain natriuretic peptide (BNP), creatinine (Cr), ejection fraction (LVEF), history of smoking and diabetes mellitus (DM)*.

## Discussion

The principal findings of this cohort study can be summarized as follows: (1) compared with only PCI, routine TA before PCI resulted in lower all-cause mortality in the univariate analysis. However, in the multivariate analysis, TA was not an independent predictive factor given the differences in the baseline characteristics and coexistence of confounders. Hence, we were unable to conclude whether TA was associated with a decrease in mortality in the entire population. (2) In the subgroup analysis according to quartiles of D-dimer levels, treatment with only PCI in patients with high D-dimer levels was associated with higher all-cause mortality than that in patients with high D-dimer levels treated with TA+PCI. Moreover, multivariate logistic regression analysis revealed a remarkable inverse relationship between TA and all-cause mortality in patients with D-dimer levels >1.5 mg/L, which proved that TA was an independent predictive factor for high D-dimer levels.

A crucial hallmark of acute STEMI is the occlusion of the culprit vessel with an intracoronary thrombus (1, 2, 11, 18). In several randomized controlled trials, TA has been shown to be effective in reducing the coronary thrombus burden and improving microvascular perfusion compared with only PCI for acute STEMI during primary PCI (19–24).

Elevated levels of specific inflammatory and oxidative stress markers have been observed in patients with a high thrombus burden, leading to impaired myocardial reperfusion in patients with STEMI (25). Especially, in patients with diabetes, the increased thrombogenicity observed in type 2 diabetes mellitus has been associated with higher glycoxidative stress, as quantified by plasma hemoglobin A1C levels and oxidative response (26). In our original cohort, the TA + PCI group had higher blood glucose levels, indicating that STEMI patients with hyperglycemia may have a higher thrombus burden, which is consistent with the results obtained by D'Onofrio et al. (27). Their study indicated that the miR33/SIRT1 pathway was involved in the increased pro-inflammatory and procoagulant status of coronary thrombosis in such patients (27). Furthermore, studies have pointed out that the mechanism of hyperglycemia leading to a high thrombus burden in patients with STEMI may include a pro-inflammatory burden. Patients in the hyperglycemic STEMI group had higher levels of pro-inflammatory cytokines [tumor necrosis factor-α (TNF-α)] (28). However, our study demonstrated that there was no significant difference in C-reactive protein levels in the patients at baseline. Whether TNF-α can be used as an indicator to verify the effect of the patients' inflammation levels on thrombosis requires further research. Therefore, it is essential to strictly treat patients with hyperglycemia; rigorous blood glucose control may increase the regenerative ability of ischemic myocardium (29), and optimal target glycemic levels and treatment regimens in patients with acute coronary syndrome (ACS) need further study (30). In patients with normal glucose tolerance undergoing PCI, there are also some factors related to disease progression. The AIRE study found that adiponectin and insulin resistance were associated with restenosis and independently related to ischemic heart disease (31). Although the findings were interesting, future studies are required due to the relatively small sample size. The TAPAS study (7) demonstrated improvement in long-term clinical outcomes with TA compared with those with PCI alone for acute STEMI during primary PCI. Two other small randomized trials showed that TA was associated with significantly less left ventricular remodeling and lower end-systolic and end-diastolic left ventricular volumes than PCI alone (22, 23). Rheolytic thrombectomy has also been reported to be safe and effective for treating proximal deep venous thrombosis (DVT) (32). To our knowledge, however, conflicting results have been reported by other trials of manual TA (33) and have largely been negative (9, 10, 34–36). Both the TASTE and TOTAL trials, two large randomized controlled trials, showed that routine TA did not lower long-term clinical outcomes following PCI for STEMI. Similar to the TASTE and TOTAL trials, during long-term follow-up, our study also showed no significant differences in all-cause mortality between the TA and PCI-only groups. Although not significant, in the subgroup of patients with a high thrombus burden, there was an increase in stroke or transient ischemic attack (TIA). Routine manual thrombectomy was related to an increased risk of stroke and no-reflow phenomenon (NRP) relative to that with PCI only (37, 38). However, in our study, only four patients experienced stroke, and there were no differences in the risk of stroke and NRP between the TA + PCI and PCI-only groups. Sex and age influenced the demographics and morbidity of hospitalized elderly patients (39). Currently, techniques can be used to differentiate unidentical frailty phenotypes and to help define the optimal nursing strategy according to the specific patient needs (40). Moreover, a study showed that TA may benefit patients with STEMI in high-risk groups, such as the frail elderly population (41). Our research circumvented the influence of age or other comorbidities on the results through a multivariate analysis. As TA may benefit elderly patients more, further research is required.

D-dimer is the final product of crosslinked fibrin degradation by plasmin and indicates active thrombus formation and lysis and has been linked to thrombus burden. In addition to the diagnostic use of D-dimer for STEMI, this marker may have a potential prognosis (42). A significant increase in the level of D-dimer indicates thrombotic complications in patients with MI, which suggests that in addition to being used as a marker for early diagnosis, D-dimer is a risk factor in the development of MI complications (43–45). The prognostic information of D-dimer levels may be particularly useful in patients with STEMI. Plasma concentrations of D-dimer were shown to be significantly associated with thrombus burden in STEMI and were increased in ongoing or recent thrombosis (14, 46). In the present study, high D-dimer levels predicted larger myocardial infarcts, possibly due to the greater thrombosis and clot load leading to increased D-dimer levels. We hypothesize that TA during primary PCI can lower the risk of all-cause mortality in patients with STEMI with high D-dimer levels. To support this hypothesis, we conducted an analysis by dividing patients into quartile groups based on their D-dimer levels. Our study showed that the range of D-dimer levels did influence the effect of TA on all-cause mortality, and we found that TA could decrease the primary outcome in patients with D-dimer levels higher than 1.5 mg/L. However, in patients with D-dimer levels <1.5 mg/L, TA was not helpful in improving the prognoses. This indicated that TA might be an independent predictive factor in patients with STEMI with a high D-dimer level.

TA was mainly aimed at patients with heavy thrombus burden, and the connection between thrombi and D-dimer levels has also been mentioned. Therefore, when we grouped patients into quartiles of D-dimer levels, TA was linked with different results for patients with different thrombus burdens. This may explain the different outcomes between the overall population and the grouped population in the multivariate analysis. Q4 represented a group of patients with the highest thrombus burden, so they could benefit most from TA, while the first three groups may benefit less because of their lighter thrombus load.

Overall, in this study, we inferred that the effect of TA was cumulatively associated with D-dimer levels in patients with STEMI undergoing PCI. Accordingly, our data provide additional considerations for physicians in deciding if TA in patients undergoing primary PCI is beneficial.

## Limitations and strengths

Some limitations of our study should be considered. First, the trial was a single-center, non-randomized, observational study with 1,295 patients, which had limited power to investigate the interaction of TA and D-dimer levels with the clinical outcomes. We may not have been able to exclude the residual confounders despite performing extensive adjustments. Second, the findings of our study may be potentially limited by ascertainment biases related to unmeasured or hidden confounders. It was not clear in the Q3 group (0.91 < D-dimer ≤ 1.50) whether TA before PCI surgery would improve postoperative outcomes, which may be explained by the interference of other unknown confounders, and the effect of TA on patients with a D-dimer level within this range remains unknown. Third, some prespecified subgroup comparisons, such as patients with stroke, were based on a rather small number of individuals. Therefore, type II errors cannot be excluded. Our research also has several strengths. First, we assessed the effect of TA at different D-dimer levels through the D-dimer quartile level. According to this, we got a more comprehensive result instead of just setting a cutoff value to divide patients into high and low D-dimer groups, and this aspect could enrich the conclusions drawn by our research. Second, our study had a low loss ratio of follow-up rate, which would help us to obtain a more accurate judgment.

## Conclusions

The findings of our real-world study were also consistent with the 2017 ESC recommendation, which reported that routine manual TA during PCI for STEMI did not improve clinical outcomes in the study population. However, patients with STEMI with a high D-dimer level may benefit from TA during primary PCI.

## Data Availability Statement

The raw data supporting the conclusions of this article will be made available by the authors, without undue reservation.

## Ethics Statement

The studies involving human participants were reviewed and approved by Ethics Committee in Clinical Research of the First Affiliated Hospital of Wenzhou Medical University. The patients/participants provided their written informed consent to participate in this study. Written informed consent was obtained from the individual(s) for the publication of any potentially identifiable images or data included in this article.

## Author Contributions

HZ: the proposal of paper concept, funding acquisition, methodology, and responsible for the overall content as guarantor. L-yS: the proposal of paper concept and original draft writing. J-fL: methodology, original draft writing, and data analysis. Z-wL: follow up, validation, and data supervision. C-xC: data supervision and project administration. S-qL: follow up and draft editing. L-lD: data supervision. XQ: data supervision and analysis. ZG: project administration. Y-hH and J-xC: follow up. S-tK: software. All authors listed have made a substantial, direct and intellectual contribution to the work, and approved it for publication.

## Conflict of Interest

The authors declare that the research was conducted in the absence of any commercial or financial relationships that could be construed as a potential conflict of interest.

## Publisher's Note

All claims expressed in this article are solely those of the authors and do not necessarily represent those of their affiliated organizations, or those of the publisher, the editors and the reviewers. Any product that may be evaluated in this article, or claim that may be made by its manufacturer, is not guaranteed or endorsed by the publisher.

## Abbreviations

TA: thrombus aspiration
PCI: percutaneous coronary intervention
STEMI: ST-segment elevation myocardial infarction
HF: heart failure
HR: hazard ratio
95% CI: 95% confidence interval
TASTE: thrombus aspiration in myocardial infarction
TOTAL: A trial of routine aspiration thrombectomy with percutaneous coronary intervention (PCI) vs. PCI alone in patients with ST-segment elevation myocardial infarction (STEMI) undergoing primary PCI
ACEI: angiotensin-converting enzyme inhibitor
ARB: angiotensin-II receptor blocker
CK-MB: creatine kinase isoenzyme MB
BNP: brain natriuretic peptide
Cr: creatinine
LVEF: left ventricular ejection fraction
SD: standard deviation
TAPAS: Thrombus Aspiration during Percutaneous coronary intervention in Acute myocardial infarction Study.
